# Psychosocial effects of infertility among couples attending St. Michael’s Hospital, Jachie-Pramso in the Ashanti Region of Ghana

**DOI:** 10.1186/s13104-017-3008-8

**Published:** 2017-12-06

**Authors:** Reindolf Anokye, Enoch Acheampong, Wisdom Kwadwo Mprah, Janet Opia Ope, Tee Ndele Barivure

**Affiliations:** 1 Department of Community Health, Centre for Disability and Rehabilitation Studies, Kwame Nkrumah University of Science and Technology, Kumasi, Ghana; 2 Department of Nursing, Garden City University College, Kumasi, Ghana

**Keywords:** Infertility, Psychosocial effects, Married couples

## Abstract

**Objective:**

Infertility is a life crisis with a wide range of socio-cultural, emotional, physical and financial problems. This study sought to determine the psychosocial effects of infertility among couples attending St. Michael’s hospital, Jachie-Pramso. A descriptive study design was adopted using a quantitative approach. A simple random technique was used to select 100 respondents and a structured closed ended questionnaire was administered to couples who visited the St. Michaels Hospital at Jachie-Pramso.

**Results:**

The study has revealed that the social effects of infertility on couples included exclusion, verbal and physical abuse, divorce as well as stigma. There is high level of despondence among couples who are considered infertile. Reliance on family members for emotional support as well as avoidance of sensitive conversations was the main coping strategies adopted by the couples to cope with their conditions. Infertility has psychological, emotional and social consequences on individuals as well as couples. Families should support infertile individuals in every way that they can so that they will not be isolated.

## Introduction

Globally, over 80 million persons are considered infertile [1]. However, infertility rates vary among different countries, with the lowest having less than 5% to over 30% amongst the highest [1]. In the UK, one out of every seven individuals are said to be infertile [2]. Infertility is significantly higher in Sub-Saharan Africa when compared to other parts of the world [3].

In some societies possession of many children is a status symbol. Allied to this is the guarantee of cheap labour hence infertility in most marriages make men marry additional wives [4]. Men with problem of infertility often get themselves involved in anti-social behaviours like alcoholism, sexual promiscuity, prostitution, smoking [5]. To combat and control the psychosocial effects of infertility, the man ends up being intolerant of the wife on things that could be resolved amicably [6].

Infertile couples are treated with contempt and dishonour by the society, which views their infertility as a punishment for some social transgressions [7]. However, in Christian homes in Iran, though the effect is there, the man sees infertility as God’s will and involves himself in praying, fasting and waiting for God’s time because of the belief that God has a special gift for him and his wife [8]. In certain European countries such as Belgium and Austria, 14% and 10% of men respectively do not want children, while in Slovenia and Latvia; this falls to below 1% for both sexes [9].

Since African marriages are based on children, infertility could lead to separation, polygamy and finally divorce. Most traditional cultures place high social values on fertility [10] particularly as a demonstration of the consummation of the marriage.

Children are deemed important in African communities due to the fact that they are the ones who carry on family names as well as keep the lineage running. They also inherit family properties and are expected to procreate to replace departed members” [11]. In the context of religion, children are seen as divine gift from God and inability to conceive may be seen as resulting from a sin or being unworthy of God’s gift [11].

In some communities in southern Nigeria, pregnancy and childbirth are prerequisites for entry into the stage of mature womanhood and childless women are insulted as “men” by their husbands and in-laws [12]. Men who failed to have children are not considered as a real man and are often insulted as “woman” [13] [14].

The Ghanaian way of life and the how children are valued, makes it difficult for one to cope with infertility. Over the years, the cause of infertility has been attributed to both the spiritual sources and physical sickness [15]. Infertility has also been attributed to witchcraft and this is backed by claims of traditional priests that one can be fertile again if certain rites are performed to appease the gods [15]. There are several other spiritual beliefs such as disobedience to God that is attributed to infertility. It is therefore believed that prayers and repentance can be the remedy [15].

Consulting a traditional birth attendant has also been linked to the likelihood that one may attribute spiritual causes to infertility as they are usually asked to pray in order to conceive [15].

In Ghana, couples who are infertile are stigmatized socially and are not allowed to play leading roles in the society. They are not allowed to be members of the ancestral world and are perceived to be ripped of the probability to live again. Infertility also has the potential of encouraging both genders to engage in sexual activities with several partners to attest to the fact that they are fertile [16]. Greater amount of value is attached to having children and therefore particular cultural norms have been created to direct how society treats childless women [17].

This study sought to determine the psychosocial effects of infertility among couples attending St. Michael’s hospital at Jachie-Pramso in Ashanti Region of Ghana.

## Main text

### Methods

The study was carried out at St. Michael’s hospital at Jachie-Pramso in the Ashanti Region of Ghana. The St. Michael’s Hospital, serves as the main referral point for health facilities within Bosomtwe District of Ashanti Region. The district has a population of 104, 471 (projected from 2010 census with a growth rate of 3.40%). The St. Michael’s Hospital, serves as the main referral point for health facilities within Bosomtwe District of Ashanti Region. The district has a population of 104, 471 (projected from 2010 census with a growth rate of 3.40%). The District Health Administration is made up of Kuntanase, Pramso, Amakom and Jachie Sub-districts. All health issues including fertility are referred to this hospital for treatment.

A descriptive study design was employed using a quantitative approach in which structured questionnaire was administered to couples who visited the St Michaels Hospital at Jachie-Pramso for the purpose of seeking treatment for infertility.

The questionnaire was entirely new and was designed to meet the objectives of the study. The questionnaire was originally designed in English but was translated to Twi and Hausa during data collection. Demographic information that was collected included Age in years, Ethnicity, Religion, Occupation and Years of Marriage. The psychosocial domains that were measured by the questionnaire included psychological effects of infertility; social effects of infertility and infertility influence on participation in social functions.

Couples in this study were those who have married for more than at least a year and never had a child or children and selection was based on mutual consent to take part on behalf of the couple.

Verbal consent was taken from respondents because a good number of them could not read or write. The process was approved by the ethics committee after explanation. A simple random sampling technique was used to select 100 respondents using Yamane [18] simplified formula.

$$n = \frac{N}{{1 + N(e)^{2} }}$$

where *n* = sample size, *N* = population size as well as *e* = level of precision. A total of 125 couples had visited the St. Michael’s hospital Jachie-Pramso within the previous 6 months for infertility treatment. When this formula was applied to the above sample, we had;

$$N = 1 2 5$$

$$1 + N (e)^{2} = 1+ 1 2 5\left( {.0 5} \right)^{ 2}$$

$$n = \frac{125}{{1 + 125 (.05)^{2} }}$$

$$n = 9 6$$

The investigators selected 95% confidence level and a level of precision of 0.5. The investigators therefore claim that there is a 95% chance that the confidence interval that was calculated contains the true population mean with a margin of error of 5%. The 95% confidence level implies that 19 out of 20 times we conduct this survey; the results would land within our margin of error. The 5% margin of error suggest that if we surveyed all 125 couples, the results could differ with a score of minus 5% or plus 5% from its original score.

A 5% non-respondent rate was assumed and therefore 4 were added to 96 to give us a sample size of 100 couples. The number 4 was added to 96 after calculating for 5% of 96 which is 4.7. A 5% non-respondents rate was selected because it was expected that in such a study at least some respondents will decline to be part of the study at some point even though they have agreed initially to be part of the study.

In choosing the respondents a set of even and odd numbers were written on pieces of papers for them to pick on each day. On a daily basis, respondents who picked even numbers were selected. Data was collected within a period of 2 months and 4 research assistants were used. Data was entered and analyzed with data software SPSS version 21.0 and the results were presented in frequency tables and percentages.

### Results

#### Demographic characteristics of respondents

The demographic characteristics of respondents used for the study are presented in Table 1. The Mean age was 29 years and out of the total number of respondents, majority (67%) were married for between 6 to 10 yearsand were Akan’s (49%). More than half of the respondents were Christians (55%).

**Table 1:** Demographic characteristics of respondents. (Source: Field survey, 2016)

Variables	Characteristics	Frequency	Percentage (%)
Age	18–23 years	18	18
	24–29 years	29	29
	30–35 years	30	30
	36–41 years	14	14
	Above 41	9	9
	Mean = 29, SD = 6.68, Max = 41, Min = 21		
Years of marriage			
	1–5 years	31	31
	6–10 years	67	67
	11–15 years	1	1
	More than 16 years	1	1
Ethnicity			
	Akan	49	49
	Ewe	17	17
	Ga/Adangme	5	5
	Gonja	11	11
	Dagomba	17	17
	Others	1	1
Religion			
	Catholic	6	6
	Christian	55	55
	Muslim	39	39
	Traditionalist	5	5
Occupation			
	Trader	46	46
	Farmer	7	7
	Apprentice	6	6
	Student	16	16
	Artisan	4	4
	Civil servant	3	3
	Unemployed	5	5
	Hair dresser	13	13

#### Psychological effects of infertility on couples

**Fig. 1:**
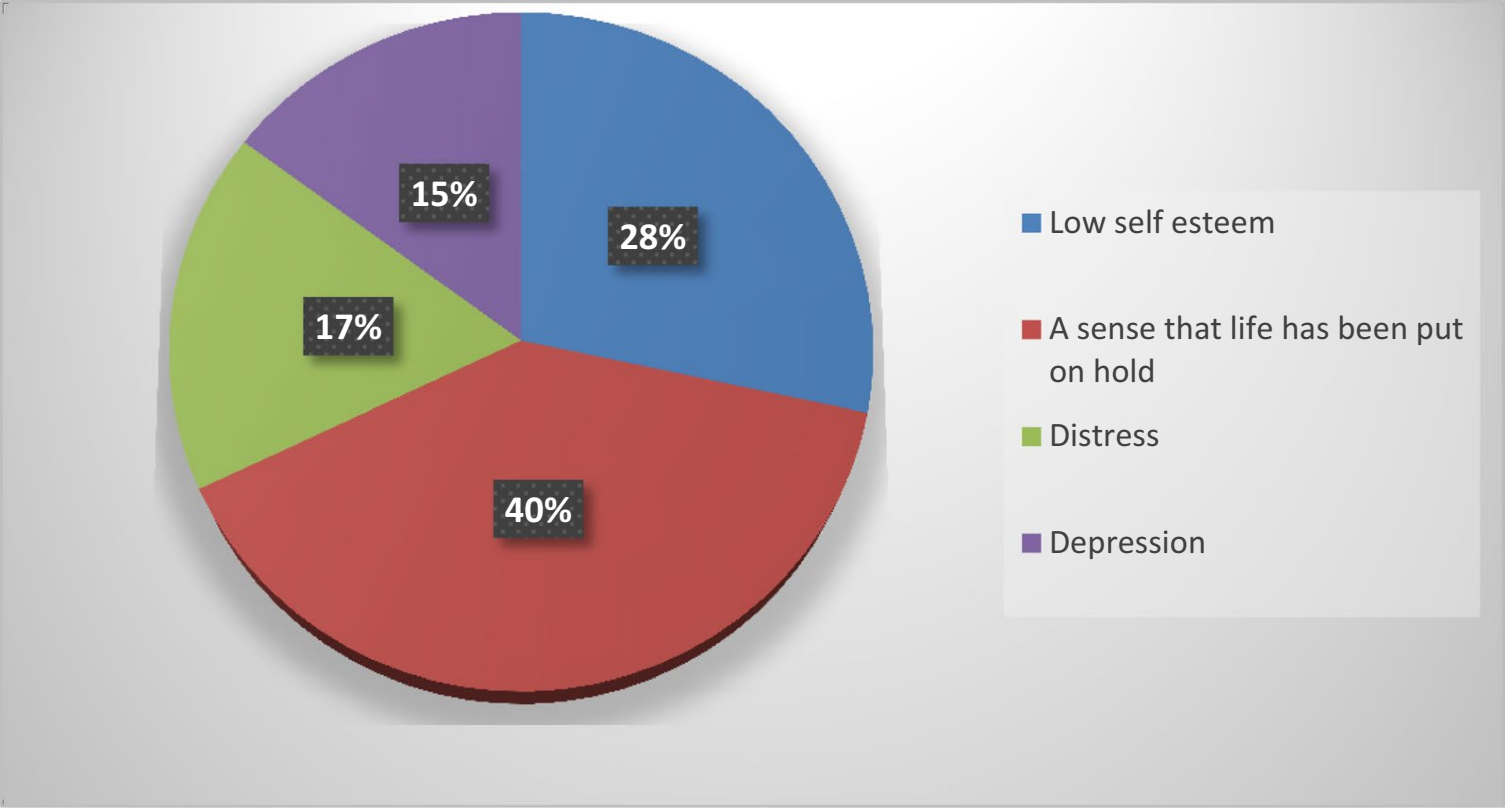
Showing the psychological effects of infertility on couples. (Source: Field work, 2016)

Figure 1 shows that 40% of the respondents indicated that the major effect of infertility is the feeling that their life has been put on hold, 28% indicated that infertility has led to low self-esteem while 17% mentioned distress and 15% mentioned depression.

#### Social effects of infertility on the lives of respondents

**Fig. 2:**
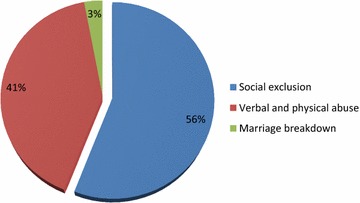
Showing social effects of infertility on the lives of respondents. (Source: Field work, 2016)

Figure 2 revealed that 56% believed infertility has led to social exclusion, 41% they are subjected to verbal and physical abuse and 3% indicated that it has led to marriage breakdown (divorce).

### Discussion

The study examined the psychosocial effects of infertility among couples attending St. Michael’s Hospital at Jachie-Pramso. Infertile couples are treated with contempt and dishonour by the society, which views their infertility as a punishment for some social transgressions [7]. This was supported by the findings of this study which revealed that living without a child has been terrible as some of them have been victims of verbal and physical abuse as well as on the verge of losing their spouse. They also indicated that they have a feeling that their life is not in motion because they do not attend any functions again and are not invited to certain programs. This is likely to affect their emotional state especially in situations where they are in the company of other couples who have children. This implies that infertility affects couples psychologically in various ways. The social consequences included infertility having affected their social life in such ways as leading to social exclusion, verbal and physical abuse and marriage breakdown. Spiritual beliefs such as disobedience to God attributed to infertility may be responsible for the way infertile couples are treated. Verbal and physical abuse meted out to them by relatives and sometimes partners may also play a role in affecting their emotions and functioning within the society. The social exclusion aspects included not attending any social functions such as marriage ceremonies because people do not invite them, even functions organized and hosted by their close relatives. This implies that in most instances, infertility leads to social exclusion.

African marriages are based on children and infertility could lead to separation, polygamy and finally divorce. Most traditional cultures place high social values on fertility particularly as a demonstration of the consummation of the marriage and as one expression of the couples’ social role. Although the need to have children is universal, it becomes even stronger in African countries where children are considered as assets and sustainable sources of income as well [19].

### Conclusion

Infertility had some level of psychological effects which were low self-esteem, frustrations and despondency on infertile couples. The social consequences of infertility included social exclusion, verbal and physical abuse as well as divorce. Also infertile couples excluded themselves from social activities because people did not invite them, even functions organized and hosted by their close relatives

#### Recommendations

1. It is recommended that, couples who are diagnosed of being infertile should form self-help groups in order for them to come together and manage their conditions effectively by providing helping hands to each other. Also, couples should support each other in the period when they are childless as this will go a long way to provide emotional support.
2. The Authorities of St. Michael Hospital should set up a special unit that will focus on providing support services such as counselling to couples who are diagnosed of being infertile and visit their facility for treatment. The unit should be staffed with clinical psychologist, medical doctors and nurses who are specialist with fertility issues with the mandate to provide psychosocial support to infertile couples.
3. It is also recommended that the Hospital authorities should collaborate with institutions such as National Commission on Civic Education (NCCE) within their district to embark on public education focusing on how to change people’s perception about infertility. Collaboration with The National Commission for Civic Education has been done in the past to educate people living with HIV/AIDS as part of the world AIDS day celebration as well as advocating for adoption of Civic Education Manual in schools.

### Limitations of the study

The study findings cannot be generalised due to the fact that it was limited to one hospital. The study population was targeted at only couples not taking into perspective the experiences of Individuals who are infertile. Also couples who had children but have lost them and want to make another but found it difficult to do so were not considered for this study.

## Abbreviation

SPSS: statistical package for social sciences
